# Diethyldithiocarbamate induces apoptosis in HHV-8-infected primary effusion lymphoma cells via inhibition of the NF-κB pathway

**DOI:** 10.3892/ijo.2011.1313

**Published:** 2011-12-20

**Authors:** TAKASHI MATSUNO, RYUSHO KARIYA, SHUICHIRO YANO, SAORI MORINO-KOGA, MANABU TAURA, MARY ANN SUICO, YUICHIRO SHIMAUCHI, SHINGO MATSUYAMA, YUKA OKAMOTO, TSUYOSHI SHUTO, HIROFUMI KAI, SEIJI OKADA

**Affiliations:** 1 Department of Molecular Medicine, Graduate School of Pharmaceutical Sciences, Global COE ‘Cell Fate Regulation Research and Education Unit’, Kumamoto University, 5-1 Oe-honmachi, Kumamoto 862-0973; 2 Division of Hematopoiesis, Center for AIDS Research, Kumamoto University, 2-2-1 Honjo, Kumamoto, 860-0811, Japan

**Keywords:** diethyldithiocarbamate, primary effusion lymphoma cells, NF-κB, proteasome inhibition

## Abstract

Primary effusion lymphoma (PEL) is a subtype of B-cell lymphoma caused by human herpes virus 8/Kaposi sarcoma-associated herpes virus (HHV-8/KSHV), which is mostly found in patients with AIDS and has poor prognosis. Nuclear factor (NF)-κB pathway is constitutively activated in HHV-8-infected PEL cells and plays a crucial role in tumorigenesis. Recently, it has been shown that diethyldithiocarbamate (DDTC), an active metabolite of disulfiram, has apoptotic activity in cancer cells. Here, we investigated the effect of DDTC on PEL using a PEL mouse model generated by intraperitoneal injection of BC-3 cells, a PEL cell line. DDTC ameliorated the symptoms of PEL in these mice, such as development of ascites, splenomegaly and increase of body weight, in comparison with PBS-treated controls. Moreover, we determined *in vitro* that DDTC suppressed the constitutively activated NF-κB pathway in BC-3 cells. Methylthiotetrazole assay revealed that the cell proliferation of various PEL cell lines was significantly suppressed by the treatment of DDTC. DDTC also induced the expression of cleaved caspase-3, an apoptosis marker, whereas the addition of Q-VD-OPh, a pan-caspase inhibitor, inhibited cell apoptosis induced by DDTC treatment. Together, our results indicated that DDTC induces apoptosis via inhibition of the NF-κB signaling pathway in HHV-8-infected PEL cells. This study suggests the potential use of DDTC as a therapeutic approach for PEL.

## Introduction

Primary effusion lymphoma (PEL) is a subtype of non-Hodgkin’s B-cell lymphoma, which is mostly found in patients with AIDS, but is also sometimes found in immunosuppressed patients such as those who have undergone organ transplantation (1,2). PEL usually presents as a lymphomatous effusion in body cavities and is caused by human herpes virus 8 (HHV-8), also known as Kaposi’s sarcoma-associated herpes virus (KSHV) (2). HHV-8 infects endothelial and B-lymphoid cells and is responsible for the development of Kaposi’s sarcoma and PEL. Despite the improved therapeutic outcome in AIDS-related lymphomas after the introduction of highly active antiretroviral therapy (HAART), PEL generally still has an extremely aggressive clinical course and the prognosis in patients with PEL is poor with a median survival of 6.2 months (3). Optimal treatment for PEL has not yet been established and development of novel therapeutic agents is needed.

A number of signaling pathways, including NF-κB, JAK/STAT and phosphoinositide 3-kinase (PI3-K) pathways, are constitutively activated and play critical roles in the survival and growth of PEL cells. HHV-8 encodes a virus Fas-associated death domain-like interleukin-1β-converting enzyme (FLICE) inhibitory protein (vFLIP) that has the ability to activate the NF-κB pathway (4,5). vFLIP has been shown to bind to the IKK complex to induce constitutive kinase activation, and as a result, PEL cells have high NF-κB pathway activity (6,7). In fact, it has been shown that inhibition of NF-κB induces apoptosis in PEL cells (8,9). These studies suggest that vFLIP-mediated NF-κB activation is essential for the survival of PEL cells and that this pathway can be a target of therapy for PEL.

Diethyldithiocarbamate (DDTC), a member of the dithiocarbamate family and metabolite of disulfiram (10,11), is a potent copper-chelating compound. Some of the known biological activities of DDTC are the inhibition of the proteasome and the induction of apoptosis in cancer cells (12). DDTC has also been used for the treatment of alcoholism, metal poisoning and HIV (13–16). Because dithiocarbamates are well-known inhibitors of NF-κB (17), we explored the possibility that DDTC could be a therapeutic agent for PEL.

In this study, we investigated the inhibitory effects of DDTC on the growth of PEL cell lines *in vitro* and *in vivo*. DDTC inhibited cell growth and induced apoptosis in HHV-8-infected PEL cells. Our findings suggest that DDTC is a promising agent for the treatment of PEL.

## Materials and methods

### Cell culture

HHV-8-infected human PEL cell lines, BCBL-1 (obtained through the AIDS Research and Reference Reagent Program, Division of AIDS, NIAID, NIH) (18), BC-1 and BC-3 (purchased from the American Type Culture Collection, Manassas, VA), and HHV-8-uninfected human cell lines, K562 (obtained from RIKEN Cell Bank, Tsukuba, Japan), were maintained in RPMI-1640 supplemented with 10% heat inactivated fetal calf serum, penicillin (100 U/ml) and streptomycin (100 μg/ml) in a humidified incubator at 37°C and 5% CO_2_.

### Animal studies

Balb/c Rag-2-deficient (Rag-2^−/−^) mice were crossed with Balb/c Jak-3-deficient (Jak-3^−/−^) mice to establish Balb/c Rag-2/Jak-3 double-deficient (Rag-2^−/−^Jak3^−/−^) mice as we have described recently (19). The mice were housed and monitored in a vivarium in compliance with the guidelines of the animal facility center of Kumamoto University. All experiments were performed according to the protocols approved by the Animal Welfare Committee of Kumamoto University (#A19–115).

### Reagents and antibodies

DDTC was purchased from Sigma-Aldrich Co. (St. Louis, MO, USA). Antibody against ubiquitinated protein (clone FK2) was purchased from BIOMOL; antibody against cleaved caspase-3 was purchased from Cell Signaling Technologies; antibodies against p65 (Sc-8008), actin (Sc-1616), γ-tubulin (Sc-7396) were purchased from Santa Cruz Biotechnology.

### Tetrazolium dye methylthiotetrazole (MTT) assay

The antiproliferative effects of DDTC against HHV-8-infected and -uninfected cell lines were measured by the MTT method. Cells were incubated in triplicate in a 96-well microculture plate in the presence of different concentrations of DDTC in a final volume of 0.1 ml for 24 h at 37°C. Subsequently, MTT (0.5 mg/ml final concentration) was added to each well. After 3 h of additional incubation, 100 μl of a solution of 0.04 N HCl in isopropanol were added to dissolve the crystal. The absorption values at 595 nm were determined with an automatic enzyme-linked immnosorbent assay (ELISA) plate reader (Multiskan, Thermo Electron, Vantaa, Finland). Values are normalized to the untreated samples.

### Annexin V assay

Apoptosis was measured by dual-labeling with the Annexin V-FITC Apoptosis Detection kit I (BD Biosciences Pharmingen). Briefly, after treatment with DDTC, cells were harvested, washed and then incubated with Annexin V-FITC and propidium iodide (PI) for 20 min in the dark, before being analyzed on an LSR II cytometer.

### Cell cycle analysis

Cells were washed with phosphate-buffered saline (PBS) and fixed with 70% ethanol. Fixed cells were washed by centrifugation in FACS washing buffer (0.5% NaN_3_, 2% FBS in PBS) and stained with PI (10 μg/ml in PBS) for 30 min in the dark. Samples were analyzed on a LSR II cytometer after nylon mesh filtration.

### Cell lysis and immunoblotting

Cells were lysed with lysis buffer (25 mM HEPES, 10 mM Na_4_P_2_O_7_·10H_2_O, 100 mM NaF, 5 mM EDTA, 2 mM Na_3_VO_4_, 1% Triton X-100). For nuclear extraction, cells were washed and resuspended in 150 μl of cold buffer containing 10 mM HEPES-KOH (pH 7.9), 10 mM KCl, 0.1 mM EDTA, 0.1 mM EGTA, 1 mM dithiothreitol and 0.5 mM phenylmethylsulfonyl fluoride (PMSF). The cells were then allowed to swell on ice for 15 min, after which 10 μl of 10% Nonidet P-40 solution was added, and the samples were vigorously vortexed for 10 sec. The homogenate was centrifuged at 15,000 × g for 1 min at 4°C. The nuclear pellet was resuspended in 50 μl of ice-cold buffer containing 20 mM HEPES-KOH (pH 7.9), 0.4 M NaCl, 1 mM EDTA, 1 mM EGTA, 1 mM dithiothreitol and 1 mM PMSF, the tube was vigorously vortexed for 15 min at 4°C. Then the nuclear extract was centrifuged at 20,000 × g for 5 min at 4°C and the clear supernatant was collected.

### Real-time RT-PCR analysis

Total RNA was isolated from cell lines using TRIzol (Invitrogen, Carlsbad, CA). Quantitative real-time RT-PCR analysis of IL-6 was carried out with Prime Script RT reagent kit and SYBR Premix Ex Taq II (Takara Bio Inc., Ohtsu, Japan) according to the manufacturer’s instructions. PCR amplifications were performed using iQ5 thermal cycler (Bio-Rad Laboratories, Inc., Hercules, CA) with the following amplification conditions: 95°C for 3 min, for 40 cycles at 95°C for 10 sec, at 55°C for 30 sec. The Ct values for each gene amplification were normalized by subtracting the Ct value calculated for β-actin (internal control). The normalized gene expression values were expressed as the relative quantity of gene-specific mRNA compared with control mRNA (fold induction). The oligonucleo-tide primers used in this study are as shown below. IL-6-Fw: 5′-GCACTGGCAGAAAACAACCT; IL-6-Rv: 5′-CAGGGGT GGTTATTGCATCT; β-actin-Fw: 5′-GCTAT C CAGGCTGTG; β-actin-Rv: 5′-TGTCACGCACGATTTCC.

### Statistical analysis

Data are presented as mean ± SE. Significance of the difference between groups was assessed with one-way ANOVA. A P<0.05 was considered statistically significant.

## Results

### Effects of DDTC on immunodeficient mice inoculated with BC-3 cells

**Figure 1:**
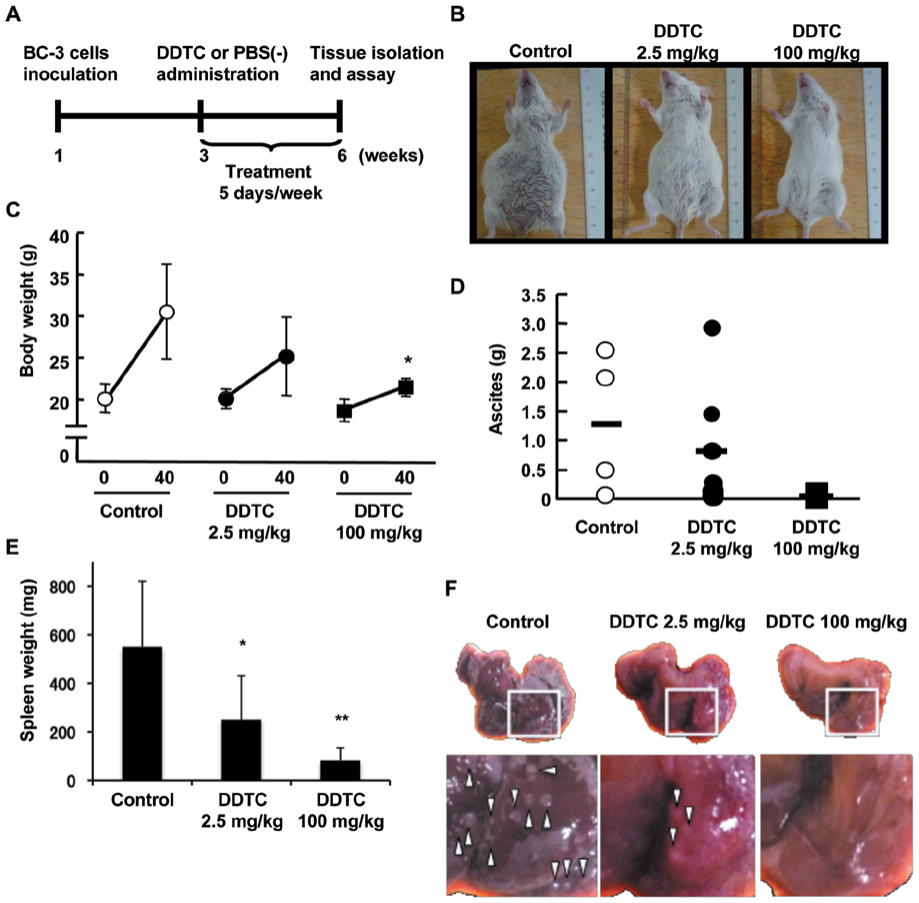
Effects of DDTC on immunodeficient mice inoculated with BC-3 cells. (A) Schematic outline of the time of BC-3 cell inoculation and DDTC treatment. (B) Representative photographs of DDTC-treated and non-treated ascites-bearing mice 6 weeks after inoculation with BC-3 cells intraperitoneally. The abdominal distention prominent in control mice was relieved by DDTC treatment. (C-E) The body weight (C), weight of ascites (D) and spleen weight (E) of the mice inoculated with BC-3 cells, untreated or treated with DDTC, was measured after the last treatment with DDTC. (F) Representative photographs of livers from DDTC-treated and non-treated mice 6 weeks after inoculation with BC-3 cells intraperitoneally. The tumor burden of livers was decreased by DDTC treatment. Data are presented as means ± SE, n=4–7 mice/group. ^*^P<0.05, ^**^P<0.01 vs. control [BC-3-inoculated, PBS(−)-treated mice], assessed by one-way ANOVA.

To evaluate the HHV-8-associated PEL response to DDTC treatment, we established a mouse model of PEL. Balb/c Rag-2^−/−^Jak3^−/−^ mice were inoculated intraperitoneally with BC-3 cells, an HHV-8-infected PEL cell line. DDTC (2.5 or 100 mg/kg) or PBS alone was administered intraperitoneally on day 21 after cell inoculation and 5 days per week thereafter for 3 weeks (Fig. 1A). It has been reported that treatment with this dose of DDTC has no toxicity in mice (20).

BC-3 cells produced massive ascites in peritoneal body cavity with evident abdominal distention, while the abdominal distention was not observed in mice that received injection of DDTC (Fig. 1B). The increase of body weight in BC-3-inoculated mice was significantly reduced by DDTC treatment in a dose-dependent manner (Fig. 1C) consistent with the difference in appearance. The alleviation of abdominal distention and decrease in body weight implied that DDTC treatment suppressed the production of ascites, therefore we examined the effect of DDTC on the amount of ascites in BC-3-inoculated mice. Although no significant decrease in the weight of ascites was observed, there was a tendency of ascites to be reduced in DDTC-treated mice in a dose-dependent manner (Fig. 1D). Previously, it has been shown that HHV-8-encoded IL-6 induces splenomegaly (21). Consistent with this report, splenomegaly was induced in mice inoculated with BC-3 cells (Fig. 1E; control). Treatment with DDTC, however, dose-dependently suppressed the development of splenomegaly (Fig. 1E). Moreover, hepatic tumorigenesis was prevented in DDTC-treated mice, but not in PBS-treated control mice (Fig. 1F). These results indicate that DDTC treatment significantly inhibits the growth of PEL cells *in vivo*.

### DDTC has proteasome inhibitory activity in HHV-8-infected and -uninfected cells

**Figure 2:**
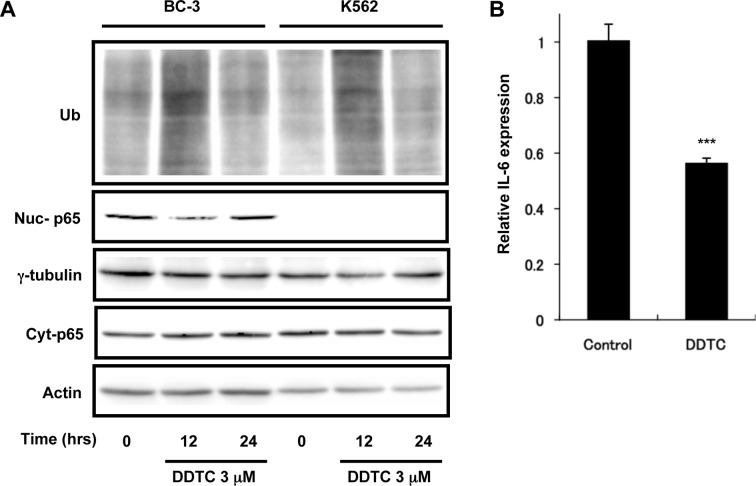
DDTC has proteasome inhibitory activity in HHV-8-infected and -uninfected cells. (A) BC-3 and K562 cells were treated with 3 μM DDTC and incubated at 37°C for 12 or 24 h. After incubation, cells were recovered and nuclear proteins were extracted. Proteins were immunoblotted using the indicated antibodies. Actin and γ-tubulin were used as protein loading control. Ub; ubiquitinated protein, Cyt; cytosolic, Nuc; nuclear. (B) BC-3 cells were treated with 3 μM DDTC and incubated at 37°C for 12 h. After incubation, the mRNA expression of IL-6 was measured by quantitative real-time PCR. IL-6 mRNA levels were normalized to the levels of β-actin. Data are presented as means ± SE (n=3). ^***^P<0.001 vs. control, assessed by one-way ANOVA.

We next determined how DDTC controls the progression of PEL. Because DDTC was shown to have proteasome inhibitory activity and induces apoptosis in prostate cancer cells (12), we investigated whether DDTC inhibits the proteasome activity in HHV-8-infected and -uninfected cells. To examine the accumulation of ubiquitinated proteins, HHV-8-infected BC-3 cells and HHV-8-uninfected K562 cells were treated for 12 or 24 h with 3 μM DDTC, an appropriate concentration for leukemia cells as determined previously (22), and cell lysates were analyzed using anti-ubiquitinated protein antibody. As shown in Fig. 2A, the accumulation of ubiquitinated proteins was observed in both BC-3 and K562 cells after treatment with DDTC for 12 h, but not for 24 h. This result suggests that DDTC has proteasome inhibitory activity irrespective of HHV-8 infection.

### DDTC suppresses the NF-κB pathway in HHV-8-infected PEL cells

DDTC is known to suppress the activation of NF-κB pathway (17), thus we next examined whether DDTC influences the NF-κB pathway in HHV-8-infected and -uninfected cells. To determine the localization and expression of p65, a subtype of NF-κB, BC-3 and K562 cells were treated with DDTC for 12 or 24 h and the nuclear extracts were subjected to immunoblotting with anti-p65 antibody. Nuclear p65 expression (Nuc-p65) was detected in control BC-3 cells, but this was reduced in DDTC-treated BC-3 cells at 12 h of treatment and returned to basal level at 24 h of treatment (Fig. 2A; Nuc-p65). The level of cytosolic p65 (Cyt-p65) was not remarkably altered (Fig. 2A). On the other hand, nuclear p65 was undetectable in K562 cells. Moreover, we examined by quantitative RT-PCR the mRNA expression of the growth factor IL-6 in BC-3 cells. IL-6 is one of the target genes of NF-κB and accelerates the cell growth in HHV-8-infected PEL cells (23). As expected, DDTC treatment for 12 h decreased the IL-6 mRNA expression (Fig. 2B), indicating that DDTC inhibited the NF-κB signaling pathway.

### DDTC induces caspase-3 dependent apoptosis in HHV-8 infected PEL cells

**Figure 3:**
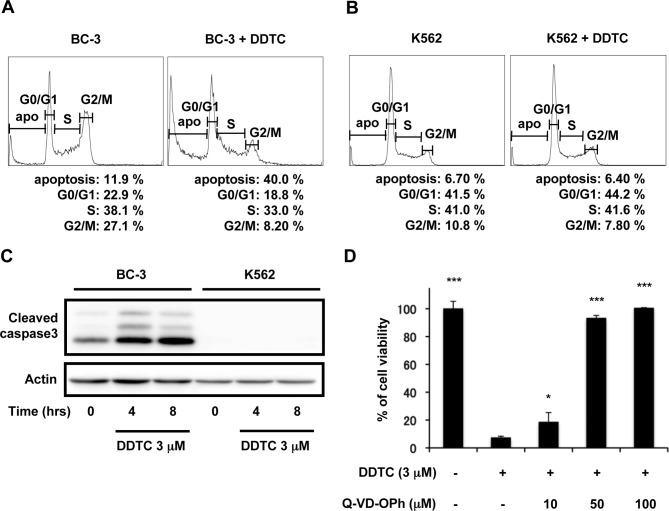
DDTC suppresses the NF-κB pathway in HHV-8-infected PEL cells. (A and B) DDTC treatment increases sub-G1 (apoptosis) populations only in HHV-8-infected cells. BC-3 (A) and K562 (B) cells were treated with 3 μM DDTC for 48 h, then cells were washed and stained with PI and analyzed for DNA content by flow cytometry. (C) BC-3 and K562 cells were treated with 3 μM DDTC and incubated at 37°C for the indicated time. After incubation, cells were recovered and the levels of cleaved caspase-3 were determined by immunoblotting. Actin was used as loading control. (D) BC-3 cells were pre-treated with caspase inhibitor Q-VD-OPh at the indicated dose for 1 h and subsequently treated with 3 μM DDTC for 24 h. After incubation, cells were recovered and subjected to MTT assay. Data are presented as mean ± SE (n=3 per group). ^*^P<0.05, ^***^P<0.001 vs. DDTC-treated cells, assessed by one-way ANOVA.

It has been demonstrated that inhibition of NF-κB induces the apoptosis of HHV-8-infected PEL cells (9,24). To determine whether inhibition of NF-κB by DDTC treatment was attributed to cell cycle arrest or apoptosis in HHV-8-infected cells, BC-3 and K562 cells were left untreated or treated with DDTC for 48 h. Cells were fixed and cell cycle fraction was determined by flow cytometry. As shown in Fig. 3A, DDTC treatment increased the population of sub-G1 cells from 12 to 40% in BC-3 cells. This increase in sub-G1 population was accompanied by reduction in G0/G1, S and G2 phases, an indication that these cells were undergoing apoptosis (25). On the other hand, there was no increase in the sub-G1 population after DDTC treatment in K562 cells (Fig. 3B). Since caspases are important mediators of apoptosis, we investigated whether DDTC treatment activates caspase-3. Caspase-3 is the main executioner protease and its activation marks a point-of-no-return in the complicated cascade of apoptosis induction (26). Cells were treated with DDTC, and cell lysates were immunoblotted with anti-cleaved caspase-3 antibody. As shown in Fig. 3C, DDTC treatment resulted in caspase-3 cleavage in BC-3 cells, but did not activate caspase-3 in K562 cells. Furthermore, pretreatment of BC-3 cells with various concentrations of Q-VD-OPh, a pan-caspase inhibitor, abrogated the cell death induced by DDTC treatment in a dose-dependent manner (Fig. 3D). These results suggest that DDTC treatment selectively induces apoptosis in BC-3 cells in caspase-3-dependent pathway.

### DDTC causes a dose-dependent inhibition of proliferation and induces apoptosis in various HHV-8-infected PEL cell lines

**Figure 4:**
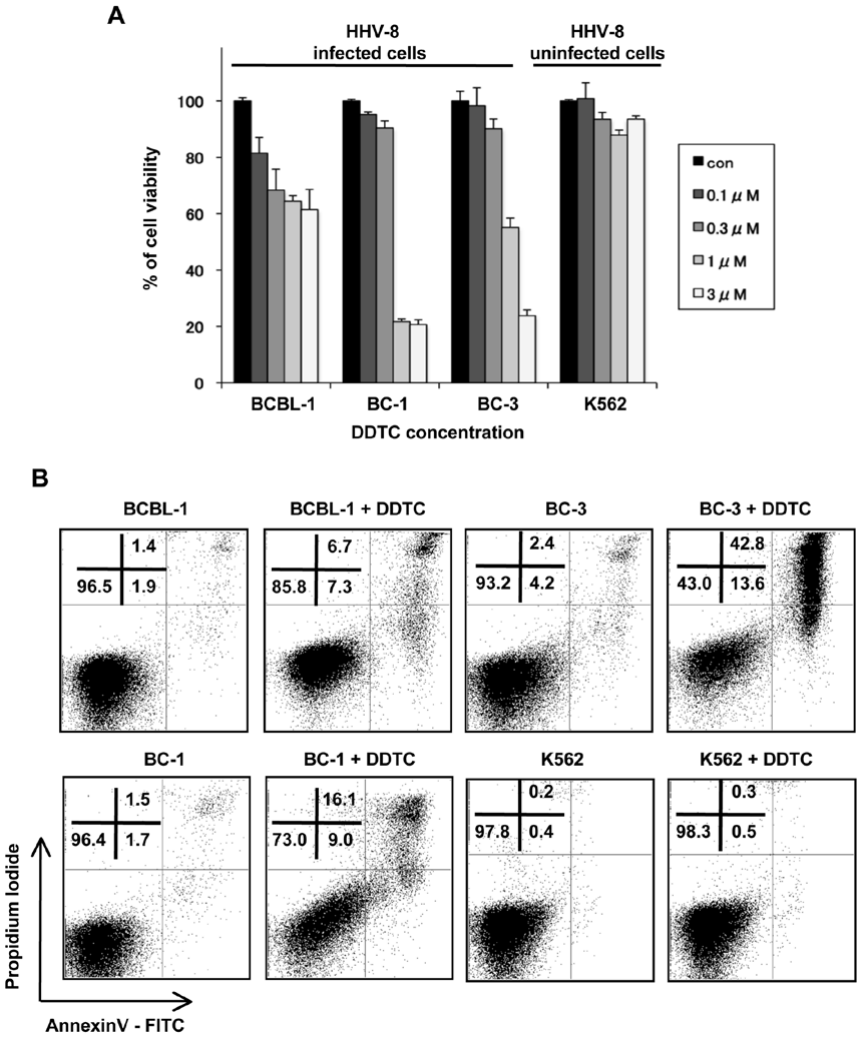
DDTC induces caspase-3-dependent apoptosis in HHV-8-infected cells. (A) HHV-8-infected cells (BCBL-1, BC-1 and BC-3) were treated with DDTC at the indicated dose and incubated at 37°C for 24 h. After incubation, cells were recovered and subjected to MTT assay. To confirm the specificity of DDTC treatment for HHV-8 infected cells, we tested the effect of DDTC on chronic myelogenous leukemia cell line K562 (HHV-8-uninfected cells). (B) Apoptosis was evaluated after treating HHV-8-infected and HHV-8-uninfected cells with 3 μM DDTC, and staining with Annexin V at 48 h. Flow cytometry profile represents Annexin V-FITC staining in x axis and PI in y axis. The number represents the percentage of early apoptotic cells (lower right quadrant) and late apoptotic cells (upper right quadrant) in each condition.

Next, we determined whether DDTC treatment leads to cell death in other HHV-8-infected cell lines. Three HHV-8-infected cell lines (BCBL-1, BC-1 and BC-3) and an HHV-8-uninfected cell line (K562) were cultured in the presence of 0.1, 0.3, 1 and 3 μM DDTC for 24 h, and MTT assay was performed. DDTC treatment decreased the viable cells in a dose-dependent manner in HHV-8-infected PEL cells (Fig. 4A). In contrast, treatment with DDTC did not affect the cell viability in K562 cells. Moreover, we carried out Annexin V binding assay followed by flow cytometry. Annexin V and propidium iodide (PI) dual staining allows separation of cells at early phases of apoptosis (Annexin V-positive, PI-negative) from those at the later stages of cell death (Annexin V-positive and PI-positive). DDTC treatment increased the population of early and late phases of apoptosis in BCBL-1, BC-1 and BC-3 cells, whereas it did not induce cell death in K562 cells (Fig. 4B). These results indicated that DDTC treatment induced apoptosis in multiple HHV-8-infected PEL cell lines thereby suppressing the growth of PEL cells.

## Discussion

In this study we showed for the first time that DDTC is a possible PEL therapeutic agent. DDTC suppressed the constitutively activated NF-κB pathway and induced caspase-3-dependent apoptosis in several PEL cell lines. DDTC also suppressed the growth of PEL cells *in vivo*. The reported use of DDTC or disulfiram as inhibitor of the canonical NF-κB pathway is due to the ability of DDTC to block the release and degradation of IκB by abolishing its phosphorylation, thereby preventing the nuclear translocation of NF-κB subunit p65 (17). It has been demonstrated that DDTC has proteasome-inhibitory and apoptotic functions (12,27), and indeed we observed that DDTC increased the accumulation of ubiquitinated proteins in BC-3 cells as well as in K562 cells (Fig. 2A). However, apoptosis was highly induced only in HHV-8-infected (BC-3) cells but not in K562 cells (Fig. 3A and B). HHV-8-infected PEL cells are known to possess a constitutively active NF-κB pathway, and its survival and proliferation are largely dependent on this pathway (8,9). Consistent with these reports, our results revealed that BC-3 cells have a high level of p65 protein expression in the nucleus that was reduced by DDTC treatment for 12 h. On the other hand, p65 protein was not detected in the nucleus of K562 (Fig. 2A), in agreement with the report that NF-κB is not constitutively expressed in K562 cells but could be stimulated by ionizing radiation (28). Thus, DDTC could suppress the abnormally activated NF-κB pathway in BC-3 cells and trigger apoptosis in these cells. These findings imply that DDTC may selectively cause apoptosis in NF-κB-dependent proliferative cells. Hence it is possible that DDTC induces apoptosis in not only PEL cell lines but also in other lymphoma cells that has abnormally activated NF-κB pathway. Indeed, we detected by MTT assay that DDTC treatment decreased the viability of Raji cells, a Burkitt lymphoma cell line (data not shown), in which NF-κB pathway is constitutively activated (29). In agreement with this finding, a recent report showed that treatment with pharmacological inhibitors of NF-κB pathway, including DDTC, reduced the growth of medulloblastoma *in vivo* (30). It is also interesting to note the study of Kanno *et al*, wherein they showed that human leukemia cell lines NALM-6 and HL-60, which have constitutively activated NF-κB signaling (31,32), are sensitive to DDTC treatment while K562 cell line was mostly unaffected by DDTC (22). The lack of apoptosis, despite the proteasomal inhibition, observed in DDTC-treated K562 cells (Figs. 2A and 3B) would suggest that proteasomal inhibition mediated by DDTC does not necessarily lead to cell death. Rather, the induction of apoptotic pathway is also contingent to the molecular constitution of the cells. This concept may be relevant when determining and assessing the potential therapeutic effects of DDTC on other cancer cells especially because accumulated studies on DDTC demonstrating its function as ubiquitin-proteasome inhibitor has fostered interest on DDTC as a possible anti-tumor agent (reviewed in refs. 27,33,34).

DDTC is a metabolite of disulfiram, which can be administrated orally and has few adverse effects (10). The dosage of DDTC used in this study (2.5 mg/kg, 100 mg/kg) has no toxicity in mice (20). The human equivalent dose of 100 mg/kg in mice is 300 mg/m^2^ according to the standard conversion table. This dose is less than 400–600 mg/m^2^, the maximally tolerated dose of DDTC in human (35). Because PEL, unlike most non-Hodgkin’s lymphomas, is relatively resistant to standard cytotoxic chemotherapy, and virtually all PEL patients succumb to the disease (36), finding a viable therapeutic drug is imperative. Although further investigations are required to determine the most effective dosage *in vivo*, disulfiram could be a new therapeutic agent for PEL with few side effects.
